# Non-*BRCA1/2* Variants Detected in a High-Risk Chilean Cohort With a History of Breast and/or Ovarian Cancer

**DOI:** 10.1200/JGO.18.00163

**Published:** 2019-05-24

**Authors:** Christina Adaniel, Francisca Salinas, Juan Manuel Donaire, Maria Eugenia Bravo, Octavio Peralta, Hernando Paredes, Nuvia Aliaga, Antonio Sola, Paulina Neira, Carolina Behnke, Tulio Rodriguez, Soledad Torres, Francisco Lopez, Claudia Hurtado

**Affiliations:** ^1^ Programa de Alto Riesgo de Cáncer de Mama y Ovario, Clínica Las Condes, Santiago, Chile

## Abstract

Little is known about the genetic predisposition to breast and ovarian cancer among the Chilean population, in particular genetic predisposition beyond *BRCA1* and *BRCA2* mutations. In the current study, we aim to describe the germline variants detected in individuals who were referred to a hereditary cancer program in Santiago, Chile.

**METHODS:**

Data were retrospectively collected from the registry of the High-Risk Breast and Ovarian Cancer Program at Clínica Las Condes, Santiago, Chile. Data captured included index case diagnosis, ancestry, family history, and genetic test results.

**RESULTS:**

Three hundred fifteen individuals underwent genetic testing during the study period. The frequency of germline pathogenic and likely pathogenic variants in a breast or ovarian cancer predisposition gene was 20.3%. Of those patients who underwent testing with a panel of both high- and moderate-penetrance genes, 10.5% were found to have pathogenic or likely pathogenic variants in non-*BRCA1/2* genes.

**CONCLUSION:**

Testing for non-*BRCA1* and -2 mutations may be clinically relevant for individuals who are suspected to have a hereditary breast or ovarian cancer syndrome in Chile. Comprehensive genetic testing of individuals who are at high risk is necessary to further characterize the genetic susceptibility to cancer in Chile.

## INTRODUCTION

In the era of precision medicine, genetic risk assessment is a superb tool with which to evaluate an individual’s underlying susceptibility to disease. Unfortunately, in low- and middle-income countries, access to genetic counseling and testing is scarce.^1,2^ In Chile, knowledge of hereditary breast and ovarian cancer (HBOC) is mainly limited to *BRCA1/2* mutations. Multiple studies report the presence of *BRCA1* and *BRCA2* mutations in HBOC families, and one study reports the presence of nine *BRCA1/2* Chilean founder mutations^3-10^; however, little is known about the relevance of moderate-penetrance variants or non-*BRCA1/2* variants in this population.

Our center in Santiago, Chile, established a high-risk program in 2008 to evaluate individuals who may have a hereditary predisposition to breast and ovarian cancer. In the current study, we aim to describe the genetic variants identified and to detail the non-*BRCA1/2* variants discovered along with the phenotype of the affected families.

## METHODS

### Study Design

Data were accessed from the registry of the High-Risk Breast and Ovarian Cancer Program at Clínica Las Condes in Santiago, Chile. This registry contains data on individuals who are referred to the program for suspicion of an HBOC syndrome on the basis of personal or family history. Data were collected from January 1, 2008, to May 31, 2018. Index case demographics, diagnosis, genetic test reports, family history, and histopathology records were abstracted.

Before July 2015, patients that met the criteria for genetic testing as defined by National Cancer Care Network (NCCN) guidelines were tested only for mutations in *BRCA1* and *BRCA2*.^11,12^ We used the most current version of the NCCN guidelines to determine eligibility at the time of testing. We performed *BRCA1* and *BRCA2* sequencing using Sanger sequencing. Depending on laboratory availability and the costs of testing at the time of participation in the registry, molecular analysis of *BRCA1* and *BRCA2* consisted of either complete sequencing of all exons and the surrounding regions of *BRCA1* and *BRCA2*, partial sequencing of select exons of *BRCA1* and *BRCA2*, or sequencing for the Ashkenazi-Jewish founder mutations in *BRCA1* and *BRCA2*. More details are included in the Data Supplement.

**CONTEXT**

**Key Objective**
The frequency of germline mutations in breast and/or ovarian cancer predisposition genes—beyond *BRCA1* and *BRCA2*—is not well reported in Chile. This study describes the pathogenic and likely pathogenic variants identified in a single institution clinical cohort with a personal and/or family history of breast and/or ovarian cancer in Santiago, Chile.
**Knowledge Generated**
Of 315 individuals studied, 17.1% had a pathogenic or likely pathogenic variant in *BRCA1* or *BRCA2*. Of those who underwent panel testing, 9.5% had a pathogenic or likely pathogenic variant in one or more of the following genes: *RAD51C*, *RAD51D*, *ATM*, *PALB2*, *CHEK2*, and *CDH1*.
**Relevance**
Given the emerging clinical relevance of pathogenic variants in moderate-penetrance cancer predisposition genes and the significant frequency of such variants in our cohort, this study highlights the importance of multigene germline testing in Chile in individuals who are suspected to be at risk for a hereditary breast or ovarian cancer syndrome.

As a result of emerging evidence for the clinical utility of testing for moderate-penetrance mutations, after July 2015 all patients who met NCCN criteria for genetic testing for HBOC—with the exception of those with a germline mutation already identified in the family—underwent genetic panel testing. The genetic panel selected was based on personal and family history. Panel testing was performed using next-generation sequencing at Clinical Laboratory Improvement Amendments–approved commercial laboratories in the United States. All variants identified using next-generation sequencing were validated by Sanger sequencing or multiplex ligation-dependent probe amplification. More details are included in the Data Supplement.

For patients with a demonstrated germline mutation in a family member, we performed single-site analysis using Sanger sequencing. Individuals assessed before July 2015 with Sanger sequencing of *BRCA1* and *BRCA2* in whom a pathogenic variant was not identified were not routinely recalled for expanded screening with next-generation sequencing.

Each commercial laboratory used its own algorithm to classify variants. Classification of variants by academic laboratories in Chile and by the Laboratory of Oncology and Molecular Genetics in Clínica Las Condes was determined using ALAMUT software (Interactive Biosoftware, Rouen, France) and consultation of the following databases: the Breast Cancer Information Core database, Kathleen Cuningham Foundation Consortium for research into Familial Breast cancer database, Universal Mutation Database, and the Leiden Open Variation Database. After 2015, the Laboratory of Oncology and Molecular Genetics also consulted the BRCA Exchange and ClinVar databases. Classification of variants in all laboratories was based on the International Agency for Research on Cancer five-tier classification scheme.

### Ethical Considerations

The ethics committee of the institution has approved the registry—adhering to the statutes of the Helsinki Declaration—used for this study. All patient information has been deidentified. The registry only contains data from patients who formally consented to participate. All patients who underwent genetic testing received pretest and post-test genetic counseling.

## RESULTS

A total of 315 individuals with a personal or family history of breast and/or ovarian cancer underwent germline genetic testing since the initiation of the registry in 2008 to May 31, 2018. All individuals who underwent genetic testing fulfilled NCCN criteria for hereditary breast and/or ovarian cancer testing. In total, 64 of 315 individuals studied (20.3%) were found to have a pathogenic (P) or likely pathogenic (LP) germline variant. Two of the individuals had two germline variants for a total of 66 P/LP variants identified, a 20.9% variant frequency. The majority of variants (81.8%) were in *BRCA1* or *BRCA2*—26 in *BRCA1* and 28 in *BRCA2* (Table 1). Those with *BRCA1/2* mutations include seven individuals with Ashkenazi-Jewish founder mutations (13.0% of all *BRCA1/2* variants).

**TABLE 1:**
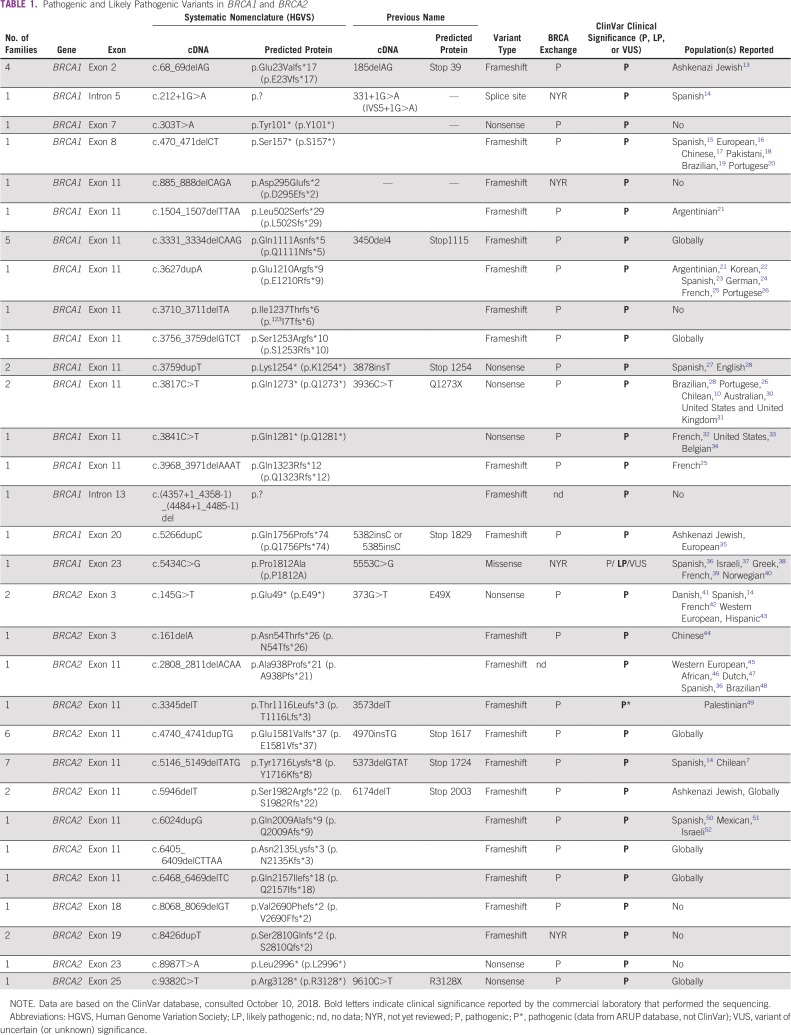
Pathogenic and Likely Pathogenic Variants in *BRCA1* and *BRCA2*

Of *BRCA1* and *BRCA2* variants reported, all of those reviewed by the Evidenced-based Network for the Interpretation of Germline Mutant Alleles are considered pathogenic variants.^53^ The only variant with conflicting interpretations of pathogenicity was the missense variant in *BRCA1*, c.5434C>G (p.Pro1812Ala). This variant has not yet been reviewed by Evidenced-based Network for the Interpretation of Germline Mutant Alleles, but has been classified in ClinVar as pathogenic, likely pathogenic, and variant of uncertain significance; it is classified as pathogenic by the Consortium of Investigators of Modifiers of *BRCA1/2*. This variant has been observed in several individuals with a personal and family history consistent with HBOC, segregating with disease in two kindreds.^36-40^ RNA and minigene assays have demonstrated that this variant causes the skipping of exon 22 in most transcripts, which leads to a truncated protein product and disrupts the second *BRCA1* C-terminal domain.^39,40^ It was not observed in approximately 6,500 individuals of European and African American ancestry in the National Heart, Lung, and Blood Institute Exome Sequencing Project. On the basis of this evidence, we consider *BRCA1* c.5434C>G to be an LP variant.

Of the 315 patients assessed, 105 were tested with genetic panels—all patients tested after July 2015 without an indication for single-site analysis. Nine of 105 individuals assessed with a panel (8.6%) had P variants in non-*BRCA1/2* genes, as classified by the commercial laboratory that performed the testing. This includes three P variants in *CHEK2*, one variant in *CDH1*, four variants in *PALB2*, and one variant in *RAD51D* (Table 2). Two LP variants were identified in non-*BRCA1/2* genes, an LP variant in *ATM*, and an LP variant in *RAD51C*. These LP variants were found in the same individual; LP variants account for 3.8% of the individuals tested with panels. More details are included in the Data Supplement.

**TABLE 2:**
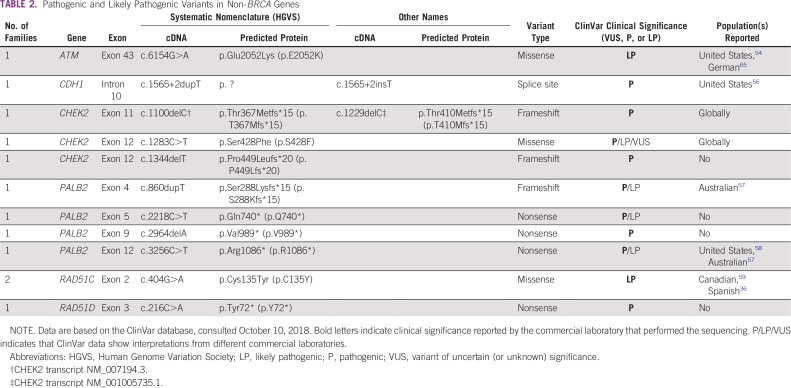
Pathogenic and Likely Pathogenic Variants in Non-*BRCA* Genes

### Detailed Review of Non-*BRCA1/2* Mutations

Given the lack of data on the presence of moderate-penetrance mutations in the Chilean population, we have detailed the clinical histories of the 11 patients in the registry who were identified as having a P or LP variant in one of the following genes: *ATM*, *CDH1*, *CHEK2*, *PALB2*, *RAD51C*, and *RAD51D*.

#### RAD51C.

**FIG 1:**
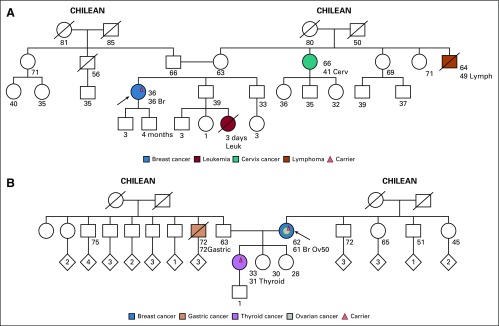
Families with a likely pathogenic (LP) variant in *RAD51C* and an LP variant in *ATM* (all ages are ages provided at the time of first contact with index case). (A) Index case, carrier of LP variant in *RAD51C* c.404G>A. No family testing has been performed to date. (B) Index case, carrier of both LP variant in *RAD51C* c.404G>A and LP variant in *ATM* c.6154G>A. The daughter is also a carrier of both LP variants. Br, breast cancer; Ov, ovarian cancer.

Two unrelated individuals were found to have the same LP variant in *RAD51C* c.404G>A. The first individual (Fig 1A) is a 36-year-old woman with triple-negative breast cancer. She has no family history of breast or ovarian cancer. *RAD51C* was included in the genetic analysis because, at the time of pretest counseling, the patient reported that her aunt had been diagnosed with ovarian cancer, which subsequently was determined to be cervix cancer. The second patient (Fig 1B) is a 62-year-old woman with both triple-negative breast cancer and papillary serous ovarian cancer diagnosed at the age of 50 years and 61 years, respectively. She has no other family history of cancer. In addition to the LP variant in *RAD51C*, she had an LP variant in *ATM* c.6154G>A. Her daughter, age 33 years and with a history of papillary thyroid cancer, was found to be a carrier of both germline LP variants.

The *RAD51C* c.404G>A (p.Cys135Tyr) variant results in a G-to-A substitution at nucleotide position 404. This alteration has been reported in Spanish and German families with breast and ovarian cancer.^60,61^ Consistent with splicing models, reverse-transcription polymerase chain reaction studies performed on RNA derived from probands in these families demonstrated that this variant results in aberrant splicing, which ultimately leads to a prematurely truncated transcript.^61^ It has not been observed in large population cohorts.^62^ In addition, it is predicted that this alteration abolishes the native splice donor site and is likely damaging and deleterious according to PolyPhen and Sorting Intolerant From Tolerant in silico analyses, respectively. On the basis of available evidence to date, this variant is considered likely pathogenic.

The *ATM* c.6154G>A variant replaces glutamic acid with lysine at codon 2052 of the ATM protein (p.Glu2052Lys). This variant is present in population databases (rs202206540; Exome Aggregation Consortium, 0.03%). It was found to be homozygous in an individual with ataxia-telangiectasia (A-T) and heterozygous in an individual affected with breast cancer.^54,55^ An experimental study that used a lymphoblastoid cell line derived from an A-T affected individual has shown that this missense change causes a defect in RNA splicing with complete loss of the ATM protein. In ClinVar, the variant is annotated as likely pathogenic for a hereditary cancer syndrome; however, we believe more epidemiologic data are required to definitively demonstrate pathogenicity.

#### CDH1.

**FIG 2:**
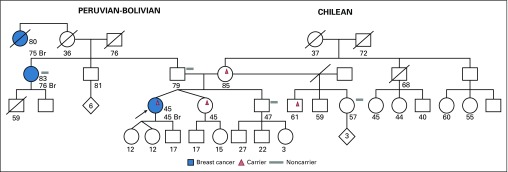
Family with a pathogenic (P) variant in *CDH1* (all ages are ages provided at the time of first contact with index case). Index case, carrier of P variant in *CDH1* c.1565+2dupT. Mother, maternal half-brother, and monozygotic twin of index case are unaffected carriers. Br, breast cancer.

The patient in whom a pathogenic variant in *CDH1* was identified (Fig 2) had been diagnosed with invasive ductal breast carcinoma. Single-site analysis demonstrated maternal inheritance; however, on the maternal side of the family, there was no history of gastric cancer or invasive lobular breast cancer. Whereas this variant has been described in some families with hereditary diffuse gastric cancer, given this patient’s family history, the significance of this intronic variant for this family is unclear.

The c.1565+2dupT intronic pathogenic mutation results from a duplication of a T nucleotide two nucleotide positions after coding exon 10 of the *CDH1* gene. This mutation has been reported in multiple individuals with hereditary diffuse gastric cancer and their affected family members.^56,63,64^ This variant is not present in population databases (Exome Aggregation Consortium, no frequency). Using the Berkeley Drosophila Genome Project and ESEfinder splice site prediction tools, it is predicted that this alteration abolishes the native splice donor site, which results in an abnormal protein or transcript that is subject to nonsense-mediated mRNA decay. This alteration is classified as a pathogenic variant in available databases.

#### CHEK2.

**FIG 3:**
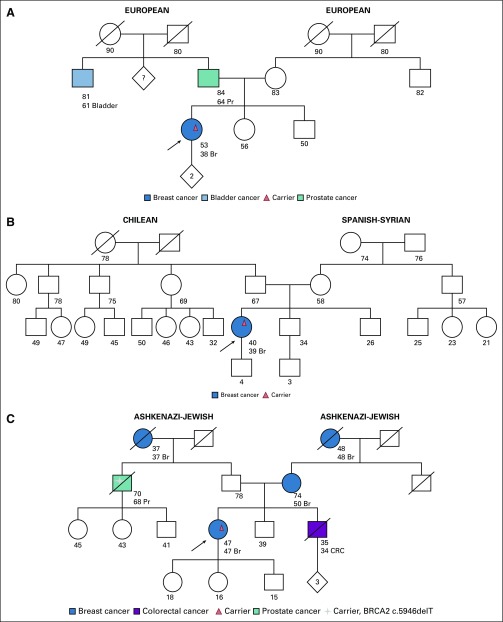
Families with pathogenic (P) variants in *CHEK2* (all ages are ages provided at the time of first contact with index case). (A) Index case, carrier of P variant in *CHEK2* c.1100delC. No family testing has been performed to date. (B) Index case, carrier of P variant in *CHEK2* c.1344delT. No family testing has been performed to date. (C) Index case, carrier of P variant in *CHEK2* c.1283C>T. Paternal uncle, carrier of P variant in *BRCA2* c.5946delT. Index case tested negative for mutations in *BRCA1* and *BRCA2*. Br, breast cancer; CRC, colorectal cancer; Pr, prostate cancer.

All three cases of *CHEK2* mutations were patients with breast cancer who were diagnosed at a young age (age 38 years, 39 years, and 47 years). The first patient had no other family history of breast cancer, but her father was diagnosed with prostate cancer at age 64 years (Fig 3A). She is of European ancestry and was found to have the *CHEK2* truncating mutation c.1100delC (p.Thr367Metfs*15). This is a well-described pathogenic variant that purportedly increases the risk of breast cancer by approximately two-fold.^65,66^

The second patient with another *CHEK2* truncating mutation, c.1344delT, was of Chilean, Spanish, and Syrian ancestry. She has no first- or second-degree relatives with cancer. She has a maternal second cousin who was diagnosed with breast cancer at age 51 years and another maternal second cousin diagnosed with pancreatic cancer at age 55 years (Fig 3B). The c.1344delT variant, located in coding exon 11 of the *CHEK2* gene, causes a translational frameshift with a predicted alternate stop codon (p.Pro449Leufs*20). This pathogenic variant has not yet been described.

The third patient, with a *CHEK2* missense mutation, c.1283C>T, is of Ashkenazi Jewish Romanian ancestry and had a family history that was notable for early-onset breast cancer of maternal lineage as well as prostate and breast cancer on the paternal side (Fig 3C). The patient’s brother died of colorectal cancer at age 35 years. Of interest, the paternal side of the family was found to have the *BRCA2* Ashkenazi Jewish founder mutation, c.5946delT, for which the patient tested negative. She also tested negative for mutations in mismatch repair genes.

In ClinVar, the *CHEK2* c.1283C>T (p.Ser428Phe) variant has conflicting interpretations of pathogenicity. This mutation is located within the kinase domain and has been demonstrated to abolish normal *CHEK2* function in yeast.^67,68^ This mutation has been reported to segregate with disease in one family tested, and it has been estimated to confer an approximate two-fold increased risk of breast cancer among Ashkenazi Jewish carrier women. It has also been identified in multiple unrelated patients with personal and family histories of breast cancer.^69,70^ On the basis of the supporting evidence, we interpret this alteration as a pathogenic variant.

#### PALB2.

**FIG 4:**
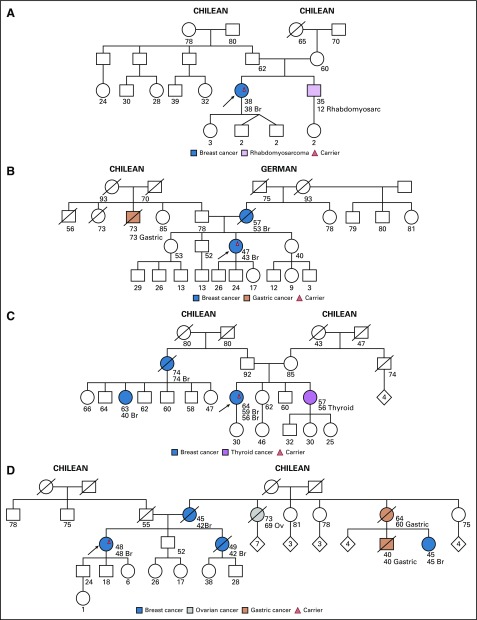
Families with pathogenic (P) variants in *PALB2* (all ages are ages provided at the time of first contact with index case). (A) Index case, carrier of P variant in *PALB2* c.860dupT. No family testing has been performed. (B) Index case, carrier of P variant in *PALB2* c.3256C>T. Sister is a carrier. (C) Index case, carrier of P variant in *PALB2* c.2964delA. No family testing has been performed to date. (D) Index case, carrier of both a P variant in *PALB2* c.2218C>T and P variant in *BRCA1* c.3817C>T. Br, breast cancer; Ov, ovarian cancer.

The four patients with *PALB2* mutations have varied family histories. The first individual, with a truncating variant (*PALB2* c.860dupT, p.Ser288Lysfs*15), was diagnosed with invasive ductal breast cancer at age 38 years. She had no family history of breast cancer; however, she has a small family with few female family members (Fig 4A). This pathogenic variant has been described previously by multiple authors.^57,71,72^

The second individual had a *PALB2* nonsense pathogenic variant, c.3256C>T (p.Arg1086*), which has been previously described in families with breast, ovarian, and pancreatic cancer. She is a 47-year-old female who was diagnosed with breast cancer at age 43 years. Her mother also had breast cancer at age 53 years. A healthy 40-year-old sister of the index case underwent single-site analysis and was found to be a carrier (Fig 4B). This pathogenic nonsense variant has also been previously reported.^57,58,71-74^

The third individual with a *PALB2* P variant was diagnosed with breast cancer at age 56 years and developed a second primary breast cancer 3 years later. She had second- and third-degree relatives with breast cancer of paternal lineage (Fig 4C). The variant detected was a nonsense mutation, c.2964delA (p.Val989*), which has been reported by various clinical laboratories and is predicted to result in loss of function.

Finally, the fourth patient with another pathogenic nonsense variant, c.2218C>T (p.Gln740*), in *PALB2* was a woman who had been diagnosed with breast cancer at age 48 years. She had multiple first-degree relatives with breast cancer (Fig 4D); however, she was found to have a concomitant *BRCA1* germline mutation, c.3817C>T. Family testing is pending.

#### RAD51D.

**FIG 5:**
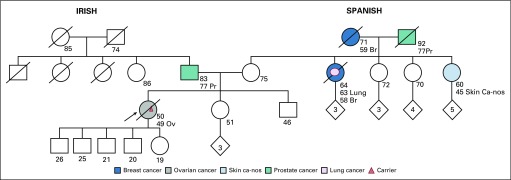
Family with a P variant in *RAD51D* (all ages are ages provided at the time of first contact with index case). Index case, carrier of P variant in *RAD51D* c.216C>A. No family testing has been performed to date. Br, breast cancer; Ov, ovarian cancer; Pr, prostate cancer.

The patient with the *RAD51D* mutation was a 50-year-old woman with stage IV papillary serous cystadenocarcinoma of the ovary who was found to have the nonsense pathogenic variant *RAD51D* c.216C>A. This variant results in a premature stop codon (p.Tyr72*) and has not yet been described. The patient’s family history was relevant for a maternal aunt with breast cancer diagnosed at age 58 years, a maternal grandmother with breast cancer diagnosed at age 59 years, and a maternal first cousin once removed with ovarian cancer diagnosed at age 45 years (Fig 5). To date, no other family members have presented for single-site analysis testing.

## DISCUSSION

Our registry represents a high-risk cohort referred for genetic testing in a single institution in Santiago, Chile. In 11 years, we studied 315 patients. In this selected cohort, 20.3% of individuals studied were determined to have a P or LP variant in a breast and/or ovarian cancer high- or moderate-penetrance gene. Of the 105 individuals who underwent genetic panel testing, 8.6% had a P variant and 3.8% had an LP variant in a non-*BRCA1/2* gene.

The two most frequent *BRCA1/2* mutations in the current study were located in exon 11 of *BRCA2*: c.4740_4741dupTG and c.5146_5149delTATG. These two mutations account for 24% of *BRCA1/2* mutations in our cohort and are considered to be Chilean founder mutations.^10^ We also report three *BRCA1/2* mutations not previously described in the literature: *BRCA1* c.3710_3711delTA, *BRCA1* c.(4357+1_4358-1)_(4484+1_4485-1)del, and *BRCA2* c.3345delT. All three of these mutations are truncating mutations, the second being the complete deletion of intron 13 in *BRCA1*. In contrast to a recent publication that reported a high cumulative frequency of nine Chilean founder mutations in *BRCA1/2*, although these variants were observed in our cohort, they only account for 36.3% of all variants.^10^

In addition to *BRCA1/2* mutations, we also identified non-*BRCA1/2* P and LP variants in six moderate–high-penetrance genes: *ATM*, *PALB2*, *CHEK2*, *CDH1*, *RAD51C*, and *RAD51D*. Other studies that evaluated these genes in the Chilean population evaluated for single-nucleotide polymorphism or common variant association with risk of disease.^75-80^ Few studies describe the presence of P or LP moderate-penetrance variants in Chilean clinical cohorts.

Individuals with moderate-penetrance variants in our study have varied family histories, some with first-degree relatives with cancers associated with the variant identified and others with multiple unaffected generations. Variability of presentation is not unexpected for individuals with moderate-penetrance mutations. Unfortunately, given the low frequency of single-site analysis in family members, we cannot definitively comment on the behavior and penetrance of these mutations in our families.

Lack of family testing reflects the barriers to genetic testing in Chile. This is a direct result of various factors: the lack of insurance coverage for genetic testing, the high cost of the exam in Chile, the lack of awareness on the part of health care providers about genetic testing criteria and the availability of the exam, and the lack of genetic counselors in Chile.^2^ To date, the Chilean public health care system provides no coverage for genetic testing of cancer predisposition genes.

Given such limitations to testing, we recognize that the population in our study represents a select group of individuals. In addition, this study is limited by the lack of homogeneity in *BRCA1* and *BRCA2* sequencing before 2015. This again reflects the limitation of resources, which early on lead to partial sequencing of *BRCA1* and *BRCA2*. Nevertheless, the utility of multigene testing and the infrequency of recurrent *BRCA1* and *BRCA2* mutations has been demonstrated in other Latin American cohorts.^81-83^ For these reasons and in light of the non-*BRCA1/2* mutations identified in this cohort, we do not advocate for the use of a limited screening panel to evaluate for Chilean founder mutations, as has been suggested by other authors.^10^

In conclusion, our understanding of the spectrum of germline mutations that may be present in the Chilean population is far from complete. We report the presence of *BRCA1/2* and non-*BRCA1/2* variants in a cohort of individuals with a personal or family history of breast and/or ovarian cancer. All variants identified are in clinically actionable genes. Recognition at the public health level of the importance of genetic testing is essential to facilitate a more systematic evaluation of patients who are at risk and a more precise understanding of the frequency of non-*BRCA1/2* mutations in the population.
